# Revised Model for the Type A Glycan Biosynthetic Pathway in *Clostridioides difficile* Strain 630Δ*erm* Based on Quantitative Proteomics of *cd0241*–*cd0244* Mutant Strains

**DOI:** 10.1021/acsinfecdis.3c00485

**Published:** 2023-11-15

**Authors:** Bart Claushuis, Arnoud H. de Ru, Sarah A. Rotman, Peter A. van Veelen, Lisa F. Dawson, Brendan W. Wren, Jeroen Corver, Wiep Klaas Smits, Paul J. Hensbergen

**Affiliations:** † Center for Proteomics and Metabolomics, Leiden University Medical Center, Leiden 2333 ZA, The Netherlands; ‡ Faculty of Infectious and Tropical Diseases, London School of Hygiene and Tropical Medicine, London WC1E 7HT, United Kingdom; § Department of Medical Microbiology, Leiden University Medical Center, Leiden 2333 ZA, The Netherlands

**Keywords:** Glycosylation, biosynthesis, enzyme, flagella, mass spectrometry, proteomics

## Abstract

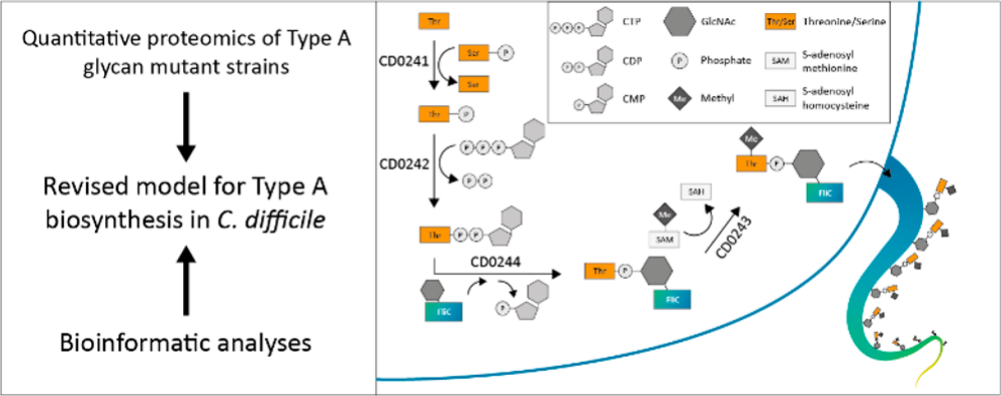

The bacterial flagellum is involved in a variety of processes including motility, adherence, and immunomodulation. In the *Clostridioides difficile* strain 630Δ*erm*, the main filamentous component, FliC, is post-translationally modified with an *O*-linked Type A glycan structure. This modification is essential for flagellar function, since motility is seriously impaired in gene mutants with improper biosynthesis of the Type A glycan. The *cd0240*–*cd0244* gene cluster encodes the Type A biosynthetic proteins, but the role of each gene, and the corresponding enzymatic activity, have not been fully elucidated. Using quantitative mass spectrometry-based proteomics analyses, we determined the relative abundance of the observed glycan variations of the Type A structure in *cd0241*, *cd0242*, *cd0243*, and *cd0244* mutant strains. Our data not only confirm the importance of CD0241, CD0242, and CD0243 but, in contrast to previous data, also show that CD0244 is essential for the biosynthesis of the Type A modification. Combined with additional bioinformatic analyses, we propose a revised model for Type A glycan biosynthesis.

Many bacteria are flagellated; i.e., they have at least one flagellum. Rotation of the flagellar filament allows directed motility toward beneficial conditions (e.g., nutrient-rich) and away from noxious environments.^1,2^ In addition, flagella mediate processes like adherence^3^ and immunomodulation.^4^ The flagellar filament is composed of repeating units of flagellin C (FliC).^5,6^ FliC O-glycosylation is essential for flagellar assembly and/or function in many species, e.g., *Helicobacter pylori* and *Campylobacter jejuni*.^7,8^ Often, the glycan structures are unique and dependent on biosynthetic pathways with unusual enzyme activities.^9,10^

In the major human gut pathogen *Clostridioides difficile*, FliC is also modified with glycan structures. In *C. difficile*, FliC glycosylation is pivotal for flagellar function because motility is seriously impaired in gene mutants with improper biosynthesis of the flagellar glycan.^11,12^ So far, two different strain-dependent glycan structures have been described, Type A and Type B,^11,13^ which only have in common the core monosaccharide that is O-linked to multiple serine and threonine residues of FliC. The Type A glycan, which is found in the *C. difficile* strain 630Δ*erm*, consists of an O-linked *N*-acetylglucosamine (GlcNAc) that is linked to *N*-methyl-l-threonine through a phosphodiester bond (Figure 1A). This structure was fully characterized by a combination of mass spectrometry (MS)^14^ and nuclear magnetic resonance spectroscopy (NMR).^11^

**Figure 1:**
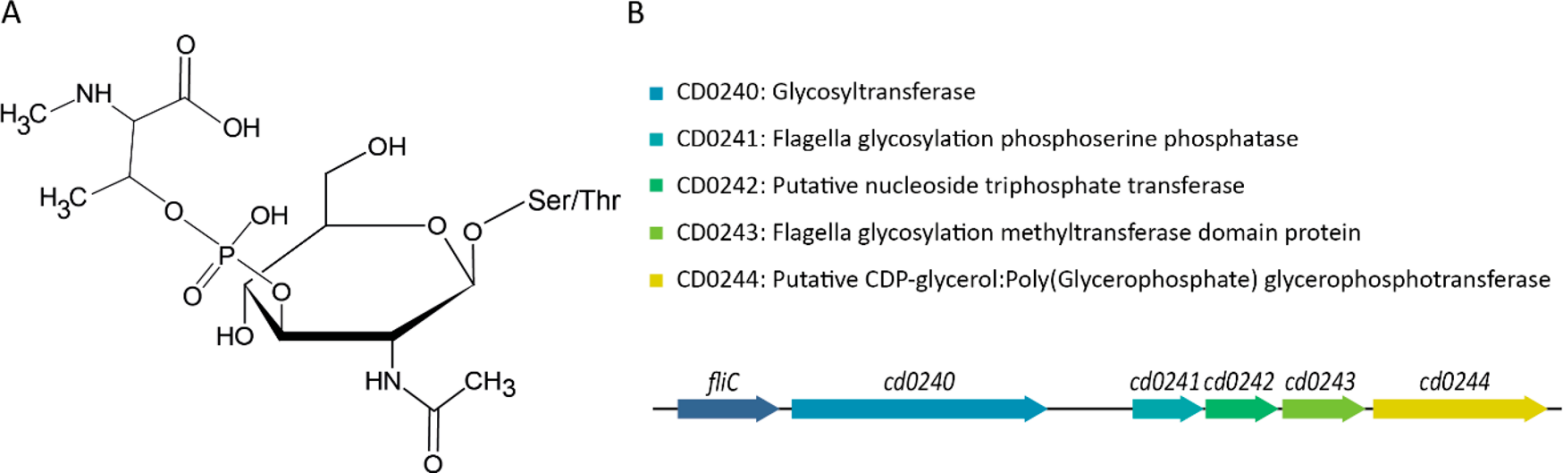
**Reported structure of the Type A glycan modification and the gene cluster responsible for its biosynthesis.** (**A**) Structure of the FliC Type A glycan. The structure consists of an O-linked GlcNAc that is linked to *N*-methyl-l-threonine through a phosphodiester bond. (**B**) The gene cluster responsible for the Type A glycan modification and the functions of the protein products as annotated in the UniProt *C. difficile* 630Δ*erm* reference proteome (Taxon ID: 272563).

In *C. difficile* 630Δ*erm*, a cluster of five genes (encoding CD0240–CD0244, Figure 1B) is linked to the biosynthesis of the Type A glycan.^11,14^ This cluster is found downstream of the *fliC* gene (*cd0239*) as part of the larger flagellar gene cluster. CD0240 is a glycosyltransferase, and disruption of this gene led to non-glycosylated FliC.^14^ The role of the other genes within the cluster is less clear, but one study looked at alterations in the Type A glycan structure in mutants with insertions in individual genes using MS analyses of FliC glycopeptides from purified flagella.^11^ In two of the mutants (*cd0241*::CT and *cd0242*::CT), flagellin was modified with only the core GlcNAc (i.e., lacking the *N*-methyl-phosphothreonine moiety). In contrast, in the *cd0243*::CT mutant strain, the Type A glycan structures lacked the *N*-methyl group on the threonine (only GlcNAc modifications were also observed), which was in line with the putative methyltransferase activity of CD0243 (Figure 1B). Surprisingly, no clear alterations in the Type A glycan structure were observed in the *cd0244*::CT strain (a mix of the full Type A glycan and GlcNAc on FliC was found), suggesting that CD0244 is redundant for Type A glycan biosynthesis. However, in the same study, it was observed that the bacterial motility in the *cd0244*::CT strain was highly impaired. The reason for this apparent inconsistency has hitherto remained elusive. Nonetheless, a model for the biosynthesis of the Type A glycan structure in *C. difficile* was proposed,^11^ in which no role for CD0244 was defined.

Interestingly, in addition to *C. difficile* (a Gram-positive bacterium), a Type A-like glycan is also found in the Gram-negative bacterium *Pseudomonas aeruginosa*, for example, in the reference strain PAO1. In this structure, the monosaccharide is a deoxyhexose which is linked to an unknown moiety through a phosphodiester bond.^15^ The similarity between the structures is also apparent from the gene cluster observed in *P. aeruginosa* (Supplemental Figure 1A). However, this cluster consists only of four genes (*pa1088*–*pa1091*, homologs of *cd0240*–*cd0243*) and lacks a gene similar to *C. difficilecd0244*.^15^ This supported the absence of an essential role for CD0244 in the model for the Type A glycan biosynthetic pathway in *C. difficile*, as described above. However, bioinformatic analyses show that *pa1091* (*fgtA*) encodes a protein with both putative glycosyltransferase activity (similar to CD0240) and phosphotransferase activity (similar to CD0244). When mapping the predicted structures of CD0240 and CD0244 to the predicted structure of PA1091 (FgtA), the enzymatic domains of these proteins align with the predicted glycosyltransferase and phosphotransferase domains of PA1091, respectively (Supplemental Figure S1B,C). Hence, this also challenges the current model for the Type A glycan biosynthesis in *C. difficile* and led us to reinvestigate the alterations of the Type A glycan on FliC in the individual *C. difficile* mutant strains. In contrast to the previous study that used qualitative analyses of FliC glycopeptides from purified flagella, we used an overall quantitative MS-based proteomics approach. Importantly, and in contrast to the previous data, we show that CD0244 is essential for full Type A glycan biosynthesis in *C. difficile*. Based on our data, we propose a revised model for the Type A glycan biosynthesis, providing testable hypotheses on the activity of individual enzymes encoded in the gene cluster.

## Results

### Relative Abundance of CD0241-CD0244 in *C. difficile* Wild-Type, Mutant, and Complemented Strains

To determine the relative abundance of the Type A biosynthetic proteins, we performed a MS-based quantitative proteomics analysis of the *C. difficile* strains from the previous study as listed in Table 1 (i.e., wild-type (WT), *cd0241*::CT, *cd0242*::CT, *cd0243*::CT, *cd0244*::CT, *cd0241*::CT comp., *cd0242*::CT comp., *cd0244*::CT comp., in duplicate) using TMTpro 16plex labeling (no complemented strain for *cd0243*::CT was available).^11^

**Table 1:** Overview of the *C. difficile* Strains Used in This Study^a^

Description	Strain	Genotype	Plasmid
Wild-Type	WKS2044	*C. difficile* strain 630Δ*erm*	None
*cd0241*::CT	WKS2047	*C. difficile* strain 630Δ*erm-cd0241*::CT	None
*cd0241*::CT complemented	WKS2048	*C. difficile* strain 630Δ*erm-cd0241*::CT	pMTL84153 with *cd0241* cloned behind the *fdx* promoter
*cd0242*::CT	WKS2049	*C. difficile* strain 630Δ*erm-cd0242*::CT	None
*cd0242*::CT complemented	WKS2050	*C. difficile* strain 630Δ*erm-cd0242*::CT	pMTL84153 with *cd0242* cloned behind the *fdx* promoter
*cd0243*::CT	WKS2051	*C. difficile* strain 630Δ*erm-cd0243*::CT	None
*cd0244*::CT	WKS2052	*C. difficile* strain 630Δ*erm-cd0244*::CT	None
*cd0244*::CT complemented	WKS2053	*C. difficile* strain 630Δ*erm-cd0244*::CT	pMTL84153 with *cd0244* cloned behind the *fdx* promoter

a Data taken from ref (11).

Overall, 2187 *C. difficile* proteins with at least two peptides were identified (Supplemental Table S1). To the best of our knowledge, this represents one of the most in-depth proteomics analyses of *C. difficile* cells. Given the aim of our study, we focused on the proteins encoded by the genes in the Type A glycan biosynthesis cluster (CD0240–CD0244), and all of them were readily identified with a high number of peptides. The data clearly showed that the levels of CD0241, CD0242, and CD0244 in the respective complemented strains were much higher than in the WT strain (Supplemental Figure S2), likely as a result of the plasmid-mediated expression under the control of a constitutive promotor from the *fdx* gene of *Clostridium pasteurianum*.^21^

Unexpectedly, the relative protein abundance of CD0241 and especially CD0244 in the respective insertion mutants did not reflect a knockout phenotype (Supplemental Figure S2 and Table S1). To rule out any unexpected issues with the strains, we performed whole genome sequencing of all *C. difficile* 630Δ*erm* strains in Table 1, which confirmed that the strains were isogenic and that the ClosTron insertions were as reported previously^11^ (data not shown). We argue that the seemingly high levels of CD0241 and especially CD0244 in their knockout strains result from the unusually high expression of these proteins in their respective complemented strains, thereby compromising the correct quantification of these proteins using TMT labels (i.e., the levels of reporter ions are outside the dynamic range for accurate TMT quantification).^22^ This was supported by the data for CD0243 in the *cd0243*::CT strain, for which no complemented strain was available, and which reflected a knockout phenotype (Supplemental Figure S2).

**Figure 2:**
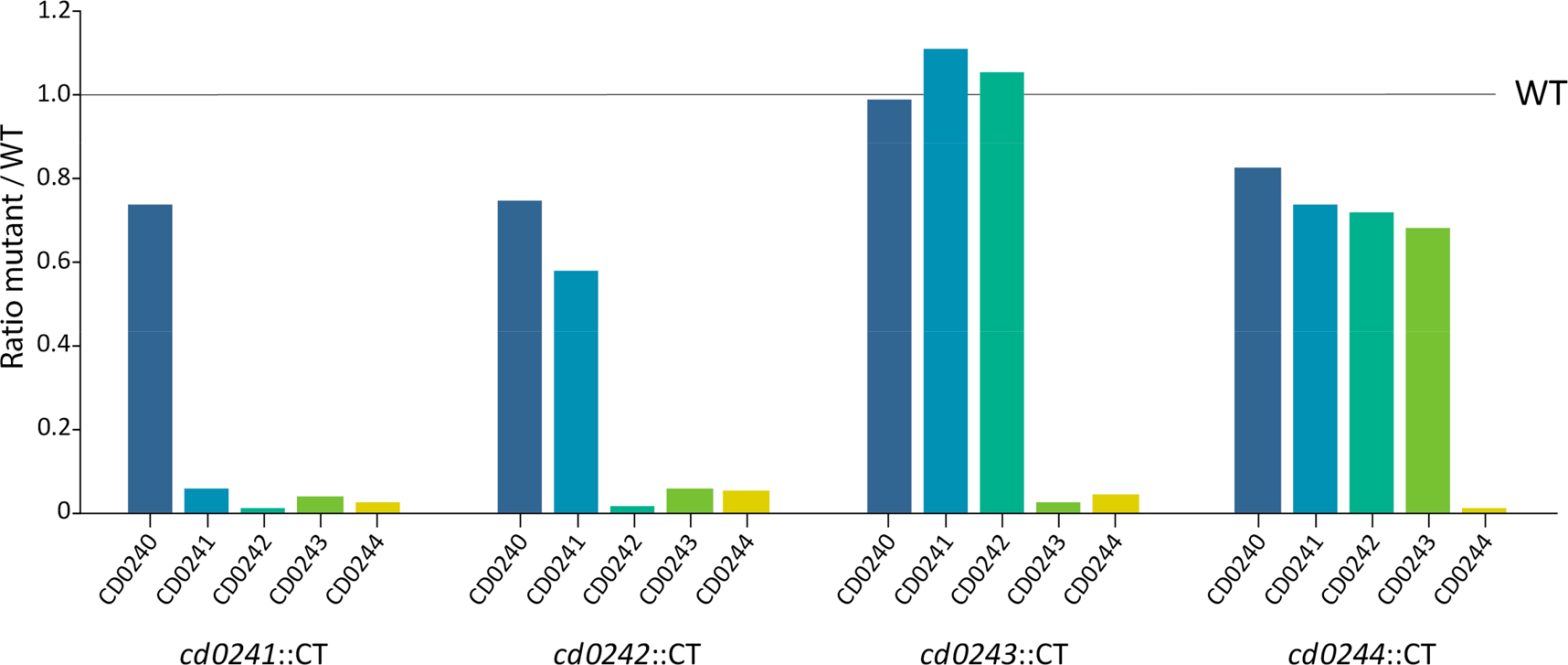
**Relative levels of the Type A biosynthetic proteins in mutants with ClosTron insertions in the individual genes.** A quantitative proteomics experiment was performed using TMTpro 15plex labeling (each strain in triplicate) and analyzed using LC-MS/MS on an Orbitrap Fusion Lumos Tribrid mass spectrometer. The protein levels of the Type A biosynthetic proteins in each of the individual gene mutant strains relative to the WT are shown. Ratios are calculated based on the average absolute abundance of a protein from three replicates per strain.

To increase the accuracy of the quantification of the proteins involved in Type A biosynthesis, a second quantitative proteomics analysis was performed in which the complemented strains were excluded. The data from this experiment confirmed the knockout phenotype of the individual insertion mutants (Figure 2). The minor residual signals can be explained by either co-isolation or impurities in the TMTpro labels; i.e., each label contains a small percentage of different isotopologues (TMT Reporter Ion Isotope Distributions for TMTpro 16plex batch WK334339, Thermo Fisher Scientific). However, Figure 2 also clearly shows an effect of the gene disruption by ClosTron mutagenesis^23^ on the downstream genes. For example, a ClosTron insertion in *cd0242* influenced protein expression from the downstream *cd0243* and *cd0244* genes yet did not influence the upstream *cd0241* gene to a similar extent. Obviously, these downstream polar effects could not be restored by complementation (Supplemental Figure S2). It is unsurprising that the ClosTron insertion caused the polar effects, given the fact that *cd0241*–*cd0244* are part of a single operon in which transcription occurs from *cd0241* throughout the rest of the genes. Since *cd0244* is the last gene of the operon, this knockout did not show disruptive polar effects on the upstream genes in the operon (Figure 2).

### Alterations of the Type A Glycan in the **cd0241*–*cd0244** Mutant Strains

To study the role of the individual genes in the **cd0241*–*cd0244** cluster on the Type A glycan biosynthesis, we explored our data from the TMTpro 16plex experiment, including all strains, for the presence of Type A glycan-modified tryptic peptides from *C. difficile* FliC (UniProt ID: Q18CX7). We focused on four different tryptic peptides of FliC that were modified with a Type A glycan structure (LLDGTSSTIR, aa 135–144; AGGTTGTDAAK, aa 191–201; TMVSSLDAALK, aa 202–212; LQVGASYGTNVSGTSNNNNEIK, aa 145–166). For each of these peptides, we concentrated on three scenarios, i.e., modification with the full Type A modification, a GlcNAc, or a Type A lacking the methyl group. MS/MS spectra corresponding to these peptides were observed in the proteomics data described above (Supplemental Table S1). However, to provide the best quantitative information, we performed additional targeted HCD MS/MS analyses of these peptides, which allowed us to sum the intensities of the TMT signals over the full peak, instead of using the TMT signals from a single MS/MS scan. In addition, this generated good-quality fragmentation spectra of our peptides of interest and their (altered) respective Type A structures.

**Figure 3:**
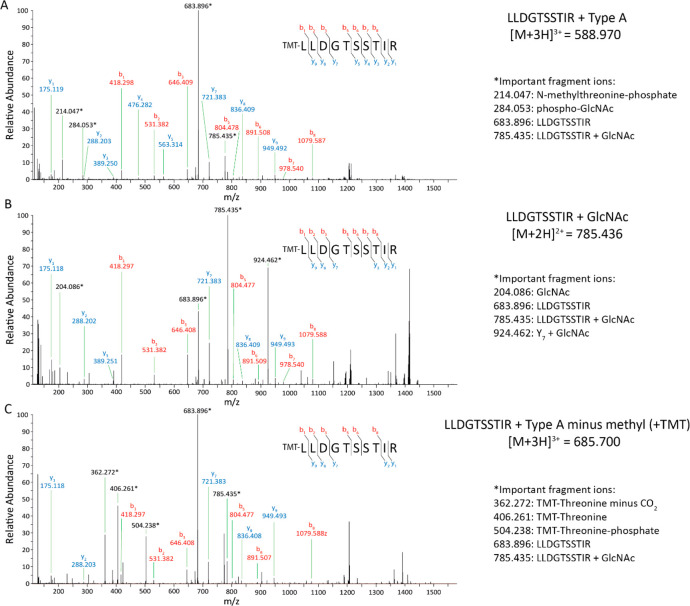
**Summed MS/MS spectra of the LLDGTSSTIR peptide displaying the Type A glycan and variants thereof.** Targeted HCD MS/MS analysis of the TMTpro 16plex labeled strains was performed. MS/MS spectra were summed over the full peak corresponding to the LLDGTSSTIR peptides displaying the complete Type A (**A**), only the GlcNAc (**B**), or Type A minus the methyl group having an extra TMT label (**C**). The theoretical precursor masses and the experimental masses of important fragment ions are shown on the right. All indicated b- and y-ions are from the unmodified TMT-labeled peptide.

The MS/MS spectrum of Type A-modified tryptic peptide LLDGTSSTIR is shown in Figure 3A. In this spectrum, Type A glycan-specific fragments at *m*/*z* 214.048 (*N*-methylthreonine-phosphate, [M+H]^+^) and 284.053 (phospho-GlcNAc, [C_8_H_15_NO_8_P]^+^) are apparent. Moreover, the major peptide fragments have lost the Type A glycan modification. For the other three peptides containing a full Type A modification, similar fragmentation characteristics were observed (Supplemental Figures S3–S5).

The MS/MS spectrum of the tryptic peptide LLDGTSSTIR modified with a single GlcNAc is shown in Figure 3B, and Supplemental Figures S3–S5 show the spectra for the other peptides. The MS/MS spectra of these species more clearly showed the GlcNAc oxonium ions, e.g., at *m*/*z* 204.087, as compared to Type A glycan-modified peptides (Figure 3A and Supplemental Figures S3–S5). The ratio of the oxonium ions at *m*/*z* 138.055 and 144.066 is consistent with a GlcNAc.^24,25^ Of note, a signal at *m*/*z* 126.055 was observed that corresponds to a GlcNAc oxonium ion, which is distinct from the 126C TMT reporter ion (*m*/*z* 126.128).

Interestingly, in the case of the absence of the *N*-methyl on the threonine as part of the Type A structure, our experimental setup would allow for TMT labeling of this extra amine group. Indeed, such FliC tryptic peptides containing the Type A glycan lacking the methyl group but with an additional TMT label were observed (Figure 3C and Supplemental Figures S3–S5). The fragmentation spectra were dominated by ions at *m*/*z* 504.239 (TMT-threonine-phosphate, [M+H]^+^ and *m*/*z* 406.262 (TMT-threonine, [M+H]^+^).

**Figure 4:**
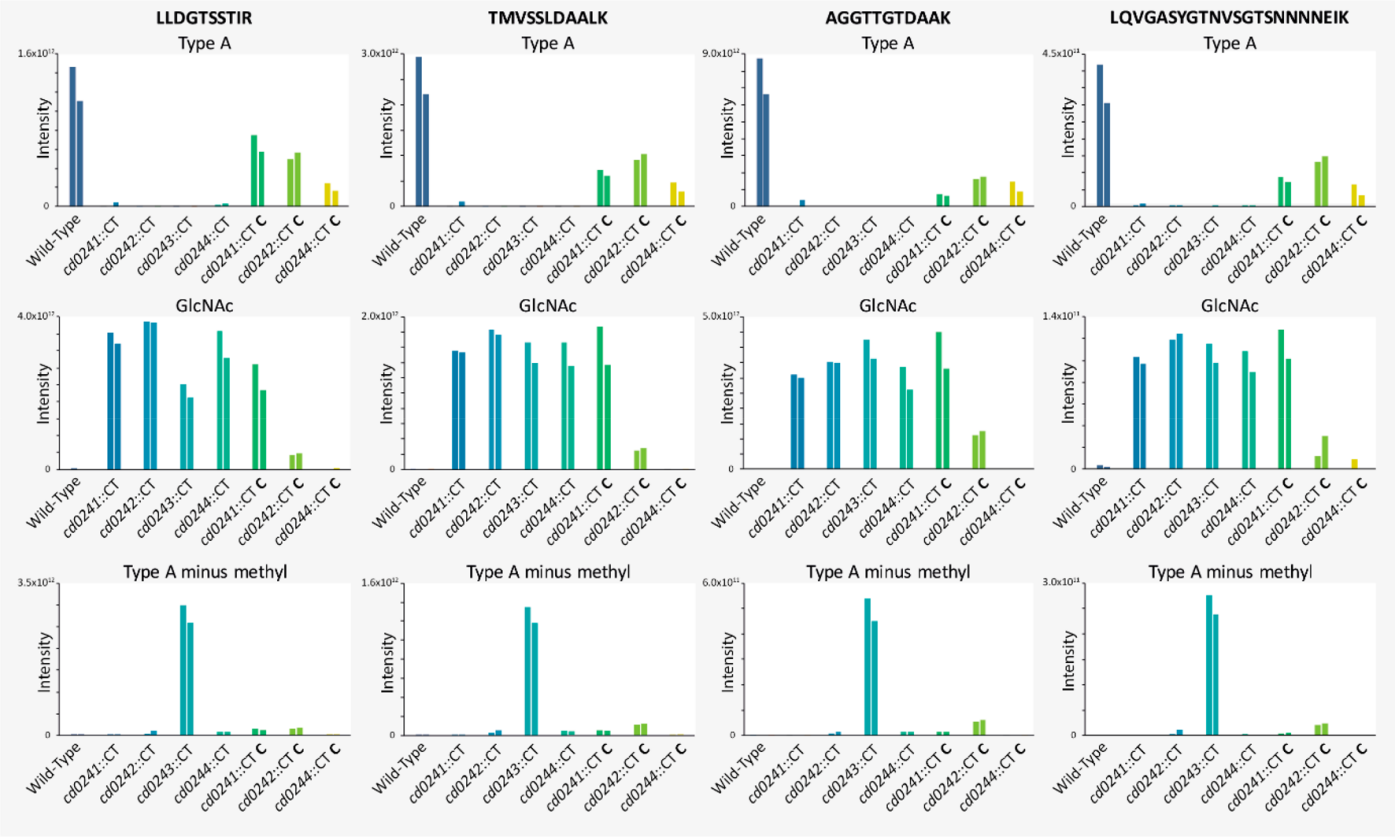
**Relative levels of peptides containing different Type A variants in individual gene mutant and complemented strains.** Targeted HCD MS/MS analysis of the TMTpro 16plex labeled strains was performed. MS/MS spectra were summed over the full peak corresponding to the peptides displaying the complete Type A, only the GlcNAc or Type A minus the methyl group, having an extra TMT label. The bars represent the absolute intensities of the TMT reporter labels for each strain, which were analyzed in duplicate. The complemented strains are indicated with a “C”.

Next, we determined the relative abundance of the differently modified FliC peptides in each of the strains from Table 1. In Figure 4, the TMT signals from the MS/MS spectra of these modified FliC tryptic peptides are depicted. In line with what was shown previously,^11^ the Type A glycan-modified FliC peptides were absent in the *cd0241*::CT, *cd0242*::CT, and *cd0243*::CT strains. However, in contrast to what was shown previously, Type A glycan-modified peptides were also absent in the *cd0244*::CT strain (Figure 4). As described above, the minor TMT signals observed for *cd0241*::CT and *cd0244*::CT in Figure 4 can be explained by impurities in the TMT labels. As expected, in addition to the WT strain, Type A glycan-modified peptides were also detected in the complemented strains, although the level of complementation varied per strain and peptide.

In line with previous data,^11^ FliC tryptic peptides with a single GlcNAc were observed in the *cd0241*::CT and *cd0242*::CT strains (Figure 4). Importantly, we clearly show that also in the *cd0244*::CT strain FliC tryptic peptides with a single GlcNAc are highly abundant, again demonstrating that the modification of FliC in this strain is radically different from that in the WT strain. FliC peptides with only GlcNAc moieties were also detected in the *cd0243*::CT strain, which is in line with what has been observed before.^11^ We propose that this is due to the polar effects on *cd0244* expression in the *cd0243*::CT strain (Figure 2).

As expected, peptides containing the Type A modification lacking the methyl group but with an additional TMT label were predominantly observed in the *cd0243*::CT strain (Figure 4). However, TMT reporter ions that could not be explained by impurities in the TMT labels were also observed in the *cd0241*::CT comp. and *cd0242*::CT comp. strains. We find it likely that this is due to a decreased efficiency in Type A biosynthesis due to the polar effects of the ClosTron insertion on *cd0243* in the *cd0241*::CT and *cd0242*::CT strains (Figure 2).

Overall, our new data not only confirm the importance of CD0241–CD0243 for full Type A glycan biosynthesis in *C. difficile* but also demonstrate that CD0244 is pivotal for full Type A glycan biosynthesis. In the *cd0244*::CT strain, the loss of the Type A glycan structure coincides with the appearance of peptides displaying only a GlcNAc.

### Revised Model for the Type A Glycan Biosynthetic Pathway in *C. difficile*

Our results are not compatible with the current model for Type A glycan biosynthesis, which did not include a role for CD0244. In the previous model, it was proposed that CD0241 catalyzes the addition of phosphate to threonine, followed by CD0242 mediating the transfer of the phosphothreonine to the GlcNAc.^11^ Finally, CD0243 catalyzes the N-methylation of the threonine, although it is unclear during which step this occurs. In addition to the lack of a role for CD0244, the previous model also did not predict how the phosphothreonine is activated as a biosynthetic intermediate that can act as a donor. Hence, the above information prompted us to formulate new hypotheses about the activities of the different enzymes in this important biosynthetic pathway.

Bioinformatic analyses show that CD0242 belongs to the family of nucleotidyl transferases, which transfer a nucleoside monophosphate moiety to an accepting molecule. For example, a Phyre2 homology search models 97% of the sequence with 99.8% confidence to GDP-mannose pyrophosphorylase (a nucleotidyl transferase) from *Leishmania donovani* (PDB: 7whs, 21% i.d.). Indeed, the *C. difficile* reference genome (strain 630) from UniProt (Taxon ID: 272563) annotates CD0242 as a nucleoside triphosphate transferase (Figure 1, ID: Q18CY2). One of the proteins that is similar to CD0242, and was mentioned in the previous study,^11^ is CTP:phosphocholine cytidylyltransferase. This cytidylyltransferase is a key enzyme in the synthesis of phosphatidylcholine referred to as the Kennedy pathway.^26^ Based on this amino acid similarity, we hypothesize that CD0242 is a CTP:phosphothreonine cytidylyltransferase that transfers CMP to phosphothreonine. The end product of the reaction is expected to be CDP-l-threonine.

CD0244, for which no role has previously been predicted, shows similarity to the CDP-glycerol:poly(glycerophosphate) glycerophosphotransferase TagF from *Staphylococcus epidermis* (Phyre2 models 77% of the sequence with 100% confidence, PDB: 3l7m, 16% i.d.), which has enzymatic activity similar to that of the phosphotransferase in the Kennedy pathway.^26^ In line with this and our new data for the *cd0244*::CT strain, we hypothesize that CD0244 is a CDP-threonine:GlcNAc threoninephosphotransferase that transfers the phosphothreonine moiety from CDP-l-threonine to the core GlcNAc on FliC.

The most challenging prediction is the role of *C. difficile* CD0241. In the previous model, it was thought to be involved in the synthesis of phosphothreonine. However, bioinformatic analyses showed the homology of CD0241 with a phosphoserine phosphatase (PSP), not a kinase. A Phyre2 homology search models 96% of the sequence with 100% confidence to the PSP from *Methanocaldococcus jannashii* (PDB: 1j97, 29% i.d.). Also, the counterpart of CD0241 in *P. aeruginosa*, PA1089, is predicted to exhibit similar activity. However, in that same organism, a different PSP-like enzyme is present that shows not only phosphatase activity but also phosphotransferase activity.^27^ This enzyme, ThrH, is a phosphoserine:homoserine phosphotransferase. Interestingly, both CD0241 and PA1089 are ThrH homologs and are predicted to adopt a fold similar to that of ThrH (Supplemental Figure S6), while many other similar PSP-like proteins display more differences in size and/or fold. In addition, homoserine is an isomer of threonine, indicating that there are only minor differences in substrates. Based on the above, we hypothesize that CD0241 (and PA1089) possesses a phosphoserine:threonine phosphotransferase activity.

**Figure 5:**
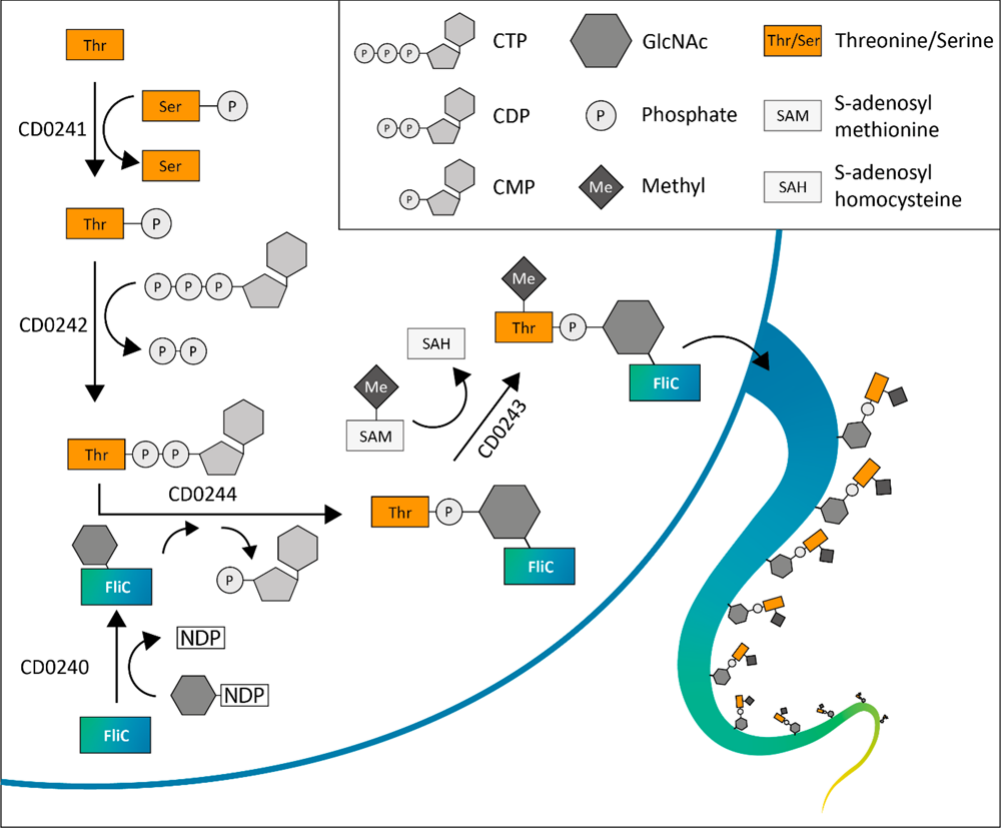
**Schematic overview of the revised model for the Type A glycan biosynthetic pathway.** First, CD0241 transfers the phosphate group from a phosphoserine to a threonine, forming phosphothreonine. Next, CD0242 transfers the phosphothreonine to CTP, thereby forming CDP-threonine, while releasing inorganic pyrophosphate. Then, CD0244 transfers the phosphothreonine to the GlcNAc moiety on FliC, which is attached by the glycosyltransferase CD0240, and this causes the release of CMP. The GlcNAc moiety on FliC is most likely donated by a nucleoside-diphosphate-GlcNAc (NDP-GlcNAc). At an unknown point during these steps, CD0243 mediates the N-methylation of threonine to form the complete Type A glycan modification. A likely donor is *S*-adenosyl methionine, which is converted to *S*-adenosyl homocysteine when donating its methyl group.

Based on our proteomics data and bioinformatic analyses, we propose a revised model for the Type A glycan biosynthesis on FliC, as shown in Figure 5. Here, CD0241 transfers the phosphate group from a phosphoserine to a threonine, forming phosphothreonine. Next, CD0242 transfers the phosphothreonine to CTP, thereby forming CDP-threonine, while releasing an inorganic pyrophosphate (PPi). Then, CD0244 transfers the phosphothreonine to the GlcNAc moiety on FliC, which is attached by the glycosyltransferase CD0240, and this causes the release of CMP. At an unknown point during these steps, CD0243 mediates the N-methylation of threonine to form the complete Type A glycan modification. A likely donor is *S*-adenosyl methionine, which is converted to *S*-adenosyl homocysteine when donating its methyl group.

## Discussion

Flagella and their roles in motility, adherence, and other host–pathogen interactions vary across different *C. difficile* lineages. For the *C. difficile* 630Δ*erm* strain, flagella are not essential for colonization of the host by the bacteria.^28−30^ However, flagellated strains display an increased fitness *in vivo*(30) and greater cecal adherence^30^ and induce a more intense inflammation^31^ than their non-flagellated counterparts. On the other hand, strains that are impaired in FliC production, the major component of the flagellar filament, have been shown to produce more exotoxins and are more virulent.^29,30^ Previous studies have shown that post-translational modification of FliC is important for flagellar function.^7,8^ Also in *C. difficile* 630Δ*erm*, disruption of several genes involved in the biosynthesis of the Type A glycan structure that is present on FliC, i.e. *cd0241*, *cd0242*, and *cd0244*, resulted in impaired mobility.^11^ Moreover, a strain that was only able to modify FliC with the core GlcNAc moiety of the Type A glycan (*cd0241*::CT) showed attenuated initial colonization and recurrence in mice.^11^ However, the proposed model for the biosynthesis of the Type A glycan defined no role for CD0244 and lacked detailed prediction on enzymatic activities and biosynthetic intermediates.^11^

Our results demonstrate a clear role for CD0244 in the biosynthesis of the Type A glycan. In the *cd0244*::CT strain, the loss of Type A coincided with the appearance of the core GlcNAc of the Type A structure. Previously, a mixed population of both structures was observed.^11^ We currently have no explanation for the discrepancy between our results and the previously reported data, especially since we used the same set of strains. However, the quantitative nature of the current study, as compared to the earlier qualitative analyses, may partially explain this. Nevertheless, the current data would explain the apparent inconsistency that was found between the impaired motility that was observed in the *cd0244*::CT strain as opposed to the absence of clear alterations in the Type A glycan structure in the earlier study.

Based on our bioinformatic analyses, we hypothesize that CD0242 mediates the synthesis of CDP-l-threonine, which would be a key biosynthetic intermediate of the Type A biosynthesis. To the best of our knowledge, CDP-l-threonine would be a so far not described cellular metabolite. However, several studies have shown the existence of amino acid residues linked to CDP in other prokaryotes, namely CDP-l-glutamine and CDP-l-serine.^32^ Furthermore, we predict CD0244 to be a CDP-threonine:GlcNAc threonine phosphotransferase. However, CD0244 also shows a similarity to UDP-*N*-acetylglucosamine 2-epimerase. This enzyme catalyzes the reversible epimerization of UDP-GlcNAc into UDP-ManNAc, the activated donor of ManNAc. Yet, this function is not supported by the data. First of all, the Type A modification has been shown to contain a GlcNAc and not a ManNAc.^11^ Second, the lack of CD0244 in the *cd0244*::CT strain does not prevent the glycosylation of FliC.

Recently, we showed that a phosphoproteomics workflow could be used to enrich Type A-modified peptides,^33^ probably due to the phospho moiety of the Type A glycan. For the current study, such an approach was not suitable because we would lose the GlcNAc-modified peptides. In our previous phosphoproteomics data, we also observed a fraction of FliC tryptic peptides that were modified with a phospho-GlcNAc.^34^ However, we have not observed the accumulation of such peptides in any of our knockout strains. Therefore, we find it likely that these peptides were the result of breakdown processes. This is supported by the fact that phospho-GlcNAc peptides could be identified in our database searches, but they all co-eluted with the full Type A-modified peptides, indicating in-source decay of the Type A peptides (data not shown). Moreover, species originated only from the WT and the complemented strains that produce the full Type A glycan, further supporting the idea that the phospho-GlcNAc moiety is not an intermediate in the biosynthesis of the Type A glycan.

Disruption of any of the genes in the **cd0241*–*cd0244** cluster using the ClosTron method completely prevents the formation of the Type A glycan. Although the levels of CD0241, CD0242, and CD0244 are unnaturally high in their respective complemented strains, this overexpression did not restore the levels of Type A-containing peptides to the WT level. For the *cd0241*::CT complemented and *cd0242*::CT complemented strains, we argue that this is due to the polar effects on the downstream genes caused by the gene insertions, which appeared to be quite strong. Nonetheless, the fact that partial complementation was possible shows that, despite the strong polar effects, active enzymes from the affected genes are still present. For the *cd0244*::CT strain, no disruptive polar effects on the upstream genes in the cluster were observed, which was also apparent from the lack of peptides with a single GlcNAc in the *cd0244*::CT complemented strain. Still, we found lower levels of Type A-modified peptides in the *cd0244*::CT complemented strain as compared to the WT strain. This, however, might be explained by low levels of FliC itself in the *cd0244*::CT complemented strain (Supplemental Table S1). FliC levels in the *cd0244*::CT complemented strain appeared to be around 6 times lower compared to the WT, and if we corrected for these differences in FliC levels, the levels of Type A-modified peptides in the *cd0244*::CT complemented strain would approach the WT levels. The nature of the lower levels of FliC in this strain remains unclear. Possibly, a feedback loop is present that responds to the overexpression of *cd0244* in the complemented strain.

## Conclusion

Based on quantitative proteomics and bioinformatic analyses, we propose a revised model for the biosynthesis of the Type A glycan modification on FliC in *C. difficile* and predict enzymatic activities for each of the involved proteins. Further experiments using these enzymes should shed more light on their activities. Our findings and model for post-translational glycan modification of flagellin in *C. difficile* will be relevant to the similar locus in *P. aeruginosa* PA01 and other bacterial species with similar flagellin modifications.

## Methods

### Bacterial Strains and Growth Conditions

The *C. difficile* strains used in this study are listed in Table 1(11) and were cultured at 37 °C in a Don Whitley A55 HEPA anaerobic workstation. The cells were grown in brain heart infusion (BHI, Oxoid) broth supplemented with 5 g/L yeast extract (BHIY) or on BHIY agar plates. When appropriate, 15 μg/mL of thiamphenicol was added.

### Sample Preparation for the Quantitative Proteomics Analysis of *C. difficile* Strains

Single colonies of *C. difficile* were picked and were precultured for 24 h in 5 mL prereduced BHIY. Next, the precultures were used to inoculate 5 mL of prereduced BHIY broth at a starting OD_600_ of 0.05, and cells were grown for 16 h. Then, cells were pelleted by centrifugation (3220*g*, 20 min, 4 °C). Pellets were resuspended in 1 mL of ice-cold PBS and washed three times (8000*g*, 5 min, 4 °C). After the last wash, pellets were resuspended in 1 mL of ST lysis buffer (5% SDS, 0.1 M Tris-HCl pH 7.5). Tubes were incubated for 20 min on ice prior to lysis by sonication, and cells were subsequently lysed by sonication for five bursts of 10 s with cooling on ice in between rounds. After lysis, tubes were centrifuged (15 min, 15000*g*, RT). Supernatants were transferred to new tubes and stored at −20 °C until further use.

For each strain, 100 μg of protein in 100 μL of ST buffer was used as the starting material. Proteins were reduced using 5 mM TCEP for 30 min, alkylated with 10 mM iodoacetamide for 30 min, and quenched with 10 mM DTT for 15 min, all at room temperature. Proteins were precipitated by chloroform–methanol precipitation. For this, 400 μL methanol, 100 μL chloroform, and 300 μL dH_2_O were added with vortexing in between each step. Following centrifugation (21130*g*, 2 min, RT), the pellet was washed two times with 500 μL methanol. The protein pellet was subsequently resuspended in 100 μL of 40 mM HEPES pH 8.4 containing 4 μg trypsin and incubated overnight at 37 °C. Again, 4 μg trypsin was added and incubated for 3 h.

TMT labeling was performed on 10 μg of tryptic peptides using TMTpro 16plex labeling (Thermo Fisher Scientific, lot no. WK334339) for 1 h at RT. Excess TMT label was quenched with 5% hydroxylamine for 15 min at RT. The labeled peptides from each sample were mixed and freeze-dried. The peptides were resuspended in 10 mM ammonium bicarbonate pH 8.4 and separated in 12 fractions on an Agilent Eclipse Plus C18 column (2.1 × 150 mm, 3.5 μM). Half of the labeled peptides (80 μg) were injected. Mobile phase A was 10 mM ammonium bicarbonate (pH 8.4). Mobile phase B was 10 mM ammonium bicarbonate in 80% acetonitrile (pH 8.4). The gradient was as follows: 2% B, 0–5 min; 2%–90% B, 5–35 min; 90% B, 35–40 min; 90%–2% B, 40–41 min; and 2% B, 41–65 min. The 12 collection vials were rotated every 30 s during sample collection. The 12 fractions were freeze-dried and stored at −20 °C prior to LC-MS/MS analysis. Two TMT experiments were performed: one with 16 samples (16plex) and one with 15 samples (15plex). The overview of the TMTpro labels for each strain is shown in Supplemental Table S2.

### LC-MS/MS Analysis

TMT-labeled peptides were dissolved in 0.1% formic acid and subsequently analyzed by online C18 nano-HPLC MS/MS with a system consisting of an Easy nLC 1200 gradient HPLC system (Thermo, Bremen, Germany) and an Orbitrap Fusion LUMOS mass spectrometer (Thermo). Fractions were injected onto a homemade precolumn (100 μm × 15 mm; Reprosil-Pur C18-AQ 3 μm, Dr Maisch, Ammerbuch, Germany) and eluted via a homemade analytical nano-HPLC column (30 cm × 75 μm; Reprosil-Pur C18-AQ 1.9 μm). The analytical column temperature was maintained at 50 °C with a PRSO-V2 column oven (Sonation, Biberach, Germany). The gradient was run from 2% to 40% solvent B (20/80/0.1 water/acetonitrile/formic acid (FA) v/v) in 120 min. The nano-HPLC column was drawn to a tip of ∼5 μm and acted as the electrospray needle of the MS source. The LUMOS mass spectrometer (Thermo) was set to use the MultiNotch MS3-based TMT method.^16^ The MS spectrum was recorded in the Orbitrap (resolution of 120,000; *m*/*z* range of 400–1500; automatic gain control (AGC) target set to 50%; maximum injection time of 50 ms). Dynamic exclusion was after *n* = 1 with an exclusion duration of 45 s and a mass tolerance of 10 ppm. Charge states 2–5 were included. Precursors for MS2/MS3 analysis were selected using “TopSpeed” with a cycle time of 3 s. MS2 analysis consisted of collision-induced dissociation (quadrupole ion trap analysis; AGC was set to “standard”; normalized collision energy (NCE) 35; maximum injection time 50 ms). The isolation window for MS/MS was 1.2 Da. Following the acquisition of each MS2 spectrum, the MultiNotch MS3 spectrum was recorded using an isolation window for MS3 of 2 Da. Ten MS2 fragments were simultaneously selected for MS3 and fragmented by high-energy collision-induced dissociation (HCD) at 65% at a custom AGC of 200% and analyzed using the Orbitrap from *m*/*z* 120 to 500 at a maximum injection time of 105 ms at a resolution of 60,000.

To obtain more accurate ratios for selected species, a separate targeted MS2 (tMS2) run was recorded for the following peptides and their selected *m*/*z*: LLDGTSSTIR with Type A, 588.97 ([M+3H]^3+^); GlcNAc, 785.44 ([M+2H]^2+^); Type A minus methyl, 685.70 ([M+3H]^3+^); AGGTTGTDAAK with Type A, 652.67 ([M+3H]^3+^); GlcNAc, 587.66 ([M+3H]^3+^); Type A minus methyl, 749.40 ([M+3H]^3+^); TMVSSLDAALK with Type A, 714.71 ([M+3H]^3+^); GlcNAc, 649.70 ([M+3H]^3+^); Type A minus methyl, 811.44 ([M+3H]^3+^); LQVGASYGTNVSGTSNNNNEIK with Type A, 819.16 ([M+4H]^4+^); GlcNAc, 770.40 ([M+4H]^4+^); Type A minus methyl, 891.71 ([M+4H]^4+^). tMS2 spectra were recorded with a precursor isolation width of 0.7 Da at an HCD collision energy of 36% at resolution 30,000 and an AGC target “standard”. The maximum injection time was set to 54 ms. MS2 spectra of each selected species were summed.

### LC-MS/MS Data Analysis

In a post-analysis process, raw data were converted to peak lists using Proteome Discoverer version 2.5.0.400 (Thermo Electron) and submitted to the UniProt *C. difficile* 630Δ*erm* database (3752 entries) (Taxon ID: 272563) using Mascot v. 2.2.07 (www.matrixscience.com) for peptide identification. Mascot searches were performed with 10 ppm and 0.5 Da deviation for precursor and fragment mass, respectively, and trypsin was selected as enzyme specificity with a maximum of two missed cleavages. The variable modifications included Type A (ST), Type A minus methyl plus TMT (ST), HexNAc (ST), Oxidation (M), and Acetyl (protein N-term). For the TMTpro 15plex search, also phosphoHexNAc was included. The static modifications included TMTpro (N-term, K) and Carbamidomethyl (C). Peptides with an FDR < 1% based on Percolator^17^ were accepted. Quantification of peptides was performed on MS3 spectra with an SPS Mass Match threshold of 100%.

### Whole Genome Sequencing

For identity confirmation, mutant strains were subjected to whole genome sequencing according to standard procedures.^18^ In short, total genomic DNA was isolated from a single colony resuspended in PBS on a QiaSymphony platform (Qiagen). Purified DNA was sequenced on the Illumina Novaseq6000 platform with a read length of 150 bp in the paired-end mode. The resultant FASTQ files were used in a reference assembly against the *C. difficile* 630 reference genome (GenBank AM180355) in Geneious software (Biomatters Ltd.); Clostron insertions were confirmed by visual identification of clusters of nucleotide polymorphisms and computational identification of high-quality single nucleotide polymorphisms using the Find Variations/SNPs algorithm in Geneious (minimum coverage 10, minimum variant frequency 0.8).

### Bioinformatic Analyses

To search for protein homologs and predict functions, the Phyre2 Web portal^19^ and the InterPro Web site for classification of protein families (www.ebi.ac.uk/interpro/search/sequence) were used. Predicted protein structures were retrieved from the Alphafold database (alphafold.ebi.ac.uk/) or modeled using Alphafold2.^20^ Analyses of protein structures were performed in PyMOL 2.5.5.

## Data Availability

The mass spectrometry proteomics data have been deposited to the ProteomeXchange Consortium^35^ via the PRIDE^36^ partner repository with the dataset identifier PXD045152.

## Supporting Information Available

Structural comparison of CD0240, CD0244, and PA1091 (Figure S1); relative levels of Type A biosynthetic proteins from TMT 16plex (Figure S2); MS/MS spectra of Type A variants on AGGTTGTDAAK (Figure S3); MS/MS spectra of Type A variants on LQVGAYGTNVSGTSNNNNEIK (Figure S4); MS/MS spectra of Type A variants on TMVSSLDAALK (Figure S5); structural comparison of CD0241, PA1089, and ThrH (Figure S6); overview of TMTpro labeling (Table S2) (PDF)
Table S1, results from database searches of LC-MS/MS data (XLSX)

## References

[ref1] Thormann K. M.; Paulick A. Tuning the flagellar motor. Microbiology 2010, 156, 1275–1283. 10.1099/mic.0.029595-0. 20203052

[ref2] Josenhans C.; Suerbaum S. The role of motility as a virulence factor in bacteria. Int. J. Med. Microbiol. 2002, 291, 605–614. 10.1078/1438-4221-00173. 12008914

[ref3] Haiko J.; Westerlund-Wikström B. The Role of the Bacterial Flagellum in Adhesion and Virulence. Biology 2013, 2, 1242–1267. 10.3390/biology2041242. 24833223 PMC4009794

[ref4] Chaban B.; Hughes H. V.; Beeby M. The flagellum in bacterial pathogens: For motility and a whole lot more. Semin. Cell Dev. Biol. 2015, 46, 91–103. 10.1016/j.semcdb.2015.10.032. 26541483

[ref5] Nakamura S.; Minamino T. Flagella-Driven Motility of Bacteria. Biomolecules 2019, 9, 279 10.3390/biom9070279. 31337100 PMC6680979

[ref6] Wadhwa N.; Berg H. C. Bacterial motility: machinery and mechanisms. Nat. Rev. Microbiol. 2022, 20, 161–173. 10.1038/s41579-021-00626-4. 34548639

[ref7] Goon S.; Kelly J. F.; Logan S. M.; Ewing C. P.; Guerry P. Pseudaminic acid, the major modification on Campylobacter flagellin, is synthesized via the Cj1293 gene. Mol. Microbiol. 2003, 50, 659–671. 10.1046/j.1365-2958.2003.03725.x. 14617187

[ref8] Schirm M.; Soo E. C.; Aubry A. J.; Austin J.; Thibault P.; Logan S. M. Structural, genetic and functional characterization of the flagellin glycosylation process in Helicobacter pylori. Mol. Microbiol. 2003, 48, 1579–1592. 10.1046/j.1365-2958.2003.03527.x. 12791140

[ref9] Chidwick H. S.; Fascione M. A. Mechanistic and structural studies into the biosynthesis of the bacterial sugar pseudaminic acid (Pse5Ac7Ac). Org. Biomol. Chem. 2020, 18, 799–809. 10.1039/C9OB02433F. 31913385

[ref10] Salah Ud-Din A. I. M.; Roujeinikova A. Flagellin glycosylation with pseudaminic acid in Campylobacter and Helicobacter: prospects for development of novel therapeutics. Cell. Mol. Life Sci. 2018, 75, 1163–1178. 10.1007/s00018-017-2696-5. 29080090 PMC11105201

[ref11] Faulds-Pain A.; Twine S. M.; Vinogradov E.; Strong P. C. R.; Dell A.; Buckley A. M.; Douce G. R.; Valiente E.; Logan S. M.; Wren B. W. The post-translational modification of the Clostridium difficile flagellin affects motility, cell surface properties and virulence. Mol. Microbiol. 2014, 94, 272–289. 10.1111/mmi.12755. 25135277 PMC4441256

[ref12] Valiente E.; Bouché L.; Hitchen P.; Faulds-Pain A.; Songane M.; Dawson L. F.; Donahue E.; Stabler R. A.; Panico M.; Morris H. R.; Bajaj-Elliott M.; Logan S. M.; Dell A.; Wren B. W. Role of glycosyltransferases modifying type B flagellin of emerging hypervirulent Clostridium difficile lineages and their impact on motility and biofilm formation. J. Biol. Chem. 2016, 291, 25450–25461. 10.1074/jbc.M116.749523. 27703012 PMC5207246

[ref13] Bouché L.; Panico M.; Hitchen P.; Binet D.; Sastre F.; Faulds-Pain A.; Valiente E.; Vinogradov E.; Aubry A.; Fulton K.; Twine S.; Logan S. M.; Wren B. W.; Dell A.; Morris H. R. The type B flagellin of hypervirulent Clostridium difficile is modified with novel sulfonated peptidylamido-glycans. J. Biol. Chem. 2016, 291, 25439–25449. 10.1074/jbc.M116.749481. 27758867 PMC5207245

[ref14] Twine S. M.; Reid C. W.; Aubry A.; McMullin D. R.; Fulton K. M.; Austin J.; Logan S. M. Motility and flagellar glycosylation in Clostridium difficile. J. Bacteriol. 2009, 191, 7050–7062. 10.1128/JB.00861-09. 19749038 PMC2772495

[ref15] Verma A.; Schirm M.; Arora S. K.; Thibault P.; Logan S. M.; Ramphal R. Glycosylation of b-type flagellin of Pseudomonas aeruginosa: Structural and genetic basis. J. Bacteriol. 2006, 188, 4395–4403. 10.1128/JB.01642-05. 16740946 PMC1482937

[ref16] McAlister G. C.; Nusinow D. P.; Jedrychowski M. P.; Wühr M.; Huttlin E. L.; Erickson B. K.; Rad R.; Haas W.; Gygi S. P. MultiNotch MS3 enables accurate, sensitive, and multiplexed detection of differential expression across cancer cell line proteomes. Anal. Chem. 2014, 86, 7150–7158. 10.1021/ac502040v. 24927332 PMC4215866

[ref17] Käll L.; Canterbury J. D.; Weston J.; Noble W. S.; MacCoss M. J. Semi-supervised learning for peptide identification from shotgun proteomics datasets. Nat. Methods 2007, 4, 923–925. 10.1038/nmeth1113. 17952086

[ref18] Ducarmon Q. R.; van der Bruggen T.; Harmanus C.; Sanders I. M. J. G.; Daenen L. G. M.; Fluit A. C.; Vossen R. H. A. M.; Kloet S. L.; Kuijper E. J.; Smits W. K. Clostridioides difficile infection with isolates of cryptic clade C-II: a genomic analysis of polymerase chain reaction ribotype 151. Clinical Microbiol. Infect. 2023, 29, 538.e1–538.e6. 10.1016/j.cmi.2022.12.003. 36509372

[ref19] Kelley L. A.; Mezulis S.; Yates C. M.; Wass M. N.; Sternberg M. J. E. The Phyre2 web portal for protein modeling, prediction and analysis. Nat. Protoc. 2015, 10, 845–858. 10.1038/nprot.2015.053. 25950237 PMC5298202

[ref20] Mirdita M.; Schütze K.; Moriwaki Y.; Heo L.; Ovchinnikov S.; Steinegger M. ColabFold: making protein folding accessible to all. Nat. Methods 2022, 19, 679–682. 10.1038/s41592-022-01488-1. 35637307 PMC9184281

[ref21] Heap J. T.; Pennington O. J.; Cartman S. T.; Minton N. P. A modular system for Clostridium shuttle plasmids. J. Microbiol Methods 2009, 78, 79–85. 10.1016/j.mimet.2009.05.004. 19445976

[ref22] Cheung T. K.; Lee C. Y.; Bayer F. P.; McCoy A.; Kuster B.; Rose C. M. Defining the carrier proteome limit for single-cell proteomics. Nat. Methods 2021, 18, 76–83. 10.1038/s41592-020-01002-5. 33288958

[ref23] Heap J. T.; Kuehne S. A.; Ehsaan M.; Cartman S. T.; Cooksley C. M.; Scott J. C.; Minton N. P. The ClosTron: Mutagenesis in Clostridium refined and streamlined. J. Microbiol. Methods 2010, 80, 49–55. 10.1016/j.mimet.2009.10.018. 19891996

[ref24] Halim A.; Westerlind U.; Pett C.; Schorlemer M.; Rüetschi U.; Brinkmalm G.; Sihlbom C.; Lengqvist J.; Larson G.; Nilsson J. Assignment of saccharide identities through analysis of oxonium ion fragmentation profiles in LC-MS/MS of glycopeptides. J. Proteome Res. 2014, 13, 6024–6032. 10.1021/pr500898r. 25358049

[ref25] Pirro M.; Mohammed Y.; de Ru A. H.; Janssen G. M. C.; Tjokrodirijo R. T. N.; Madunić K.; Wuhrer M.; van Veelen P. A.; Hensbergen P. J. Oxonium ion guided analysis of quantitative proteomics data reveals site-specific o-glycosylation of anterior gradient protein 2 (Agr2). Int. J. Mol. Sci. 2021, 22, 5369 10.3390/ijms22105369. 34065225 PMC8160981

[ref26] Kennedy E. P.; Weiss S. B. The Function of Cytidine Coenzymes in the Biosynthesis of Phospholipides. J. Biol. Chem. 1956, 222, 193–214. 10.1016/S0021-9258(19)50785-2. 13366993

[ref27] Singh S. K.; Yang K.; Karthikeyan S.; Huynh T.; Zhang X.; Phillips M. A.; Zhang H. The thrH Gene Product of Pseudomonas aeruginosa Is a Dual Activity Enzyme with a Novel Phosphoserine:Homoserine Phosphotransferase Activity. J. Biol. Chem. 2004, 279, 13166–13173. 10.1074/jbc.M311393200. 14699121

[ref28] Tasteyre A.; Barc M. C.; Collignon A.; Boureau H.; Karjalainen T. Role of FliC and FliD flagellar proteins of Clostridium difficile in adherence and gut colonization. Infect. Immun. 2001, 69, 7937–7940. 10.1128/IAI.69.12.7937-7940.2001. 11705981 PMC98895

[ref29] Dingle T. C.; Mulvey G. L.; Armstrong G. D. Mutagenic Analysis of the Clostridium difficile Flagellar Proteins, FliC and FliD, and Their Contribution to Virulence in Hamsters. Infect. Immun. 2011, 79, 4061 10.1128/IAI.05305-11. 21788384 PMC3187235

[ref30] Baban S. T.; Kuehne S. A.; Barketi-Klai A.; Cartman S. T.; Kelly M. L.; Hardie K. R.; Kansau I.; Collignon A.; Minton N. P. The Role of Flagella in Clostridium difficile Pathogenesis: Comparison between a Non-Epidemic and an Epidemic Strain. PLoS One 2013, 8, e73026 10.1371/journal.pone.0073026. 24086268 PMC3781105

[ref31] Batah J.; Kobeissy H.; Pham P. T. B.; Denève-Larrazet C.; Kuehne S.; Collignon A.; Janoir-Jouveshomme C.; Marvaud J. C.; Kansau I. Clostridium difficile flagella induce a pro-inflammatory response in intestinal epithelium of mice in cooperation with toxins. Sci. Rep. 2017, 7, 3256 10.1038/s41598-017-03621-z. 28607468 PMC5468286

[ref32] Taylor Z. W.; Raushel F. M. Manganese-Induced Substrate Promiscuity in the Reaction Catalyzed by Phosphoglutamine Cytidylyltransferase from Campylobacter jejuni. Biochemistry. 2019, 58, 2144 10.1021/acs.biochem.9b00189. 30929435 PMC6643276

[ref33] Hensbergen P. J.; De Ru A. H.; Friggen A. H.; Corver J.; Smits W. K.; Van Veelen P. A. New insights into the type A glycan modification of Clostridioides difficile flagellar protein flagellin C by phosphoproteomics analysis. J. Biol. Chem. 2022, 298, 101622 10.1016/j.jbc.2022.101622. 35065968 PMC8861647

[ref34] Smits W. K.; Mohammed Y.; de Ru A. H.; Cordo’ V.; Friggen A. H.; van Veelen P. A.; Hensbergen P. J. Clostridioides difficile Phosphoproteomics Shows an Expansion of Phosphorylated Proteins in Stationary Growth Phase. mSphere 2022, 7, 00911-21 10.1128/msphere.00911-21. PMC8730811 34986318

[ref35] Deutsch E. W.; Bandeira N.; Sharma V.; Perez-Riverol Y.; Carver J. J.; Kundu D. J.; García-Seisdedos D.; Jarnuczak A. F.; Hewapathirana S.; Pullman B. S.; Wertz J.; Sun Z.; Kawano S.; Okuda S.; Watanabe Y.; Hermjakob H.; Maclean B.; Maccoss M. J.; Zhu Y.; Ishihama Y.; Vizcaíno J. A. The ProteomeXchange consortium in 2020: enabling “big data” approaches in proteomics. Nucleic Acids Res. 2020, 48, D1145–D1152. 10.1093/nar/gkz984. 31686107 PMC7145525

[ref36] Perez-Riverol Y.; Bai J.; Bandla C.; García-Seisdedos D.; Hewapathirana S.; Kamatchinathan S.; Kundu D. J.; Prakash A.; Frericks-Zipper A.; Eisenacher M.; Walzer M.; Wang S.; Brazma A.; Vizcaíno J. A. The PRIDE database resources in 2022: a hub for mass spectrometry-based proteomics evidences. Nucleic Acids Res. 2022, 50, D543 10.1093/nar/gkab1038. 34723319 PMC8728295

