# Supplementary material for: Revised Model for the Type A Glycan Biosynthetic Pathway in Clostridioides difficile Strain 630Δerm Based on Quantitative Proteomics of cd0241–cd0244 Mutant Strains
Source: ACS Infect Dis. 2023 Nov 15;9(12):2665–74. doi: 10.1021/acsinfecdis.3c00485 (PMC10714395; doi:10.1021/acsinfecdis.3c00485)
Supplement: Supplementary file 1 — id3c00485_si_001.pdf [file id3c00485_si_001.pdf]

Supporting information

A revised model for the Type A glycan biosynthetic pathway in *Clostridioides difficile* strain 630Δ*erm* based on quantitative proteomics of *cd0241-cd0244* mutant strains

Bart Claushuis[1], Arnoud H. de Ru[1], Sarah A. Rotman[1], Peter A. van Veelen[1], Lisa F. Dawson[2], Brendan W. Wren[2], Jeroen Corver[3], Wiep Klaas Smits[3], Paul J. Hensbergen[1*]

[1] Center for Proteomics and Metabolomics, Leiden University Medical Center, Leiden, 2333 ZA, The Netherlands

[2] Faculty of Infectious and Tropical Diseases, London School of Hygiene and Tropical Medicine, London, WC1E 7HT, United Kingdom

[3] Department of Medical Microbiology, Leiden University Medical Center, Leiden, 2333 ZA, The Netherlands

*Correspondence to:

P.J. Hensbergen

Center for Proteomics and Metabolomics

Leiden University Medical Center

PO Box 9600

2300 RC Leiden

The Netherlands

Tel.: +31-71-5266394

E-mail: P.J.Hensbergen@lumc.nl

Table of Contents

Figure S1. PA1091 from *P. aeruginosa* contains multiple domains that are similar to both CD0240 and CD0244 from *C. difficile* 630Δ*erm* S3

Figure S2. The relative levels of the Type A biosynthetic proteins in mutants with ClosTron insertions in the individual genes and their complemented strains S4

Figure S3. Summed MS/MS spectra of the AGGTTGTDAAK peptide displaying the Type A variants ... S5

Figure S4. Summed MS/MS spectra of the LQVGASYGTNVSGTSNNNNNEIK peptide displaying the Type A variants S6

Figure S5. Summed MS/MS spectra of the TMVSSLDAALK peptide displaying the Type A variants S7

Figure S6. The predicted structures of CD0241 (*C. difficile*) and PA1089 (*P. aeruginosa*) are similar to the experimentally determined structure of ThrH from *P. aeruginosa* S8

Table S2. Overview of the TMTpro labels per strain. S9

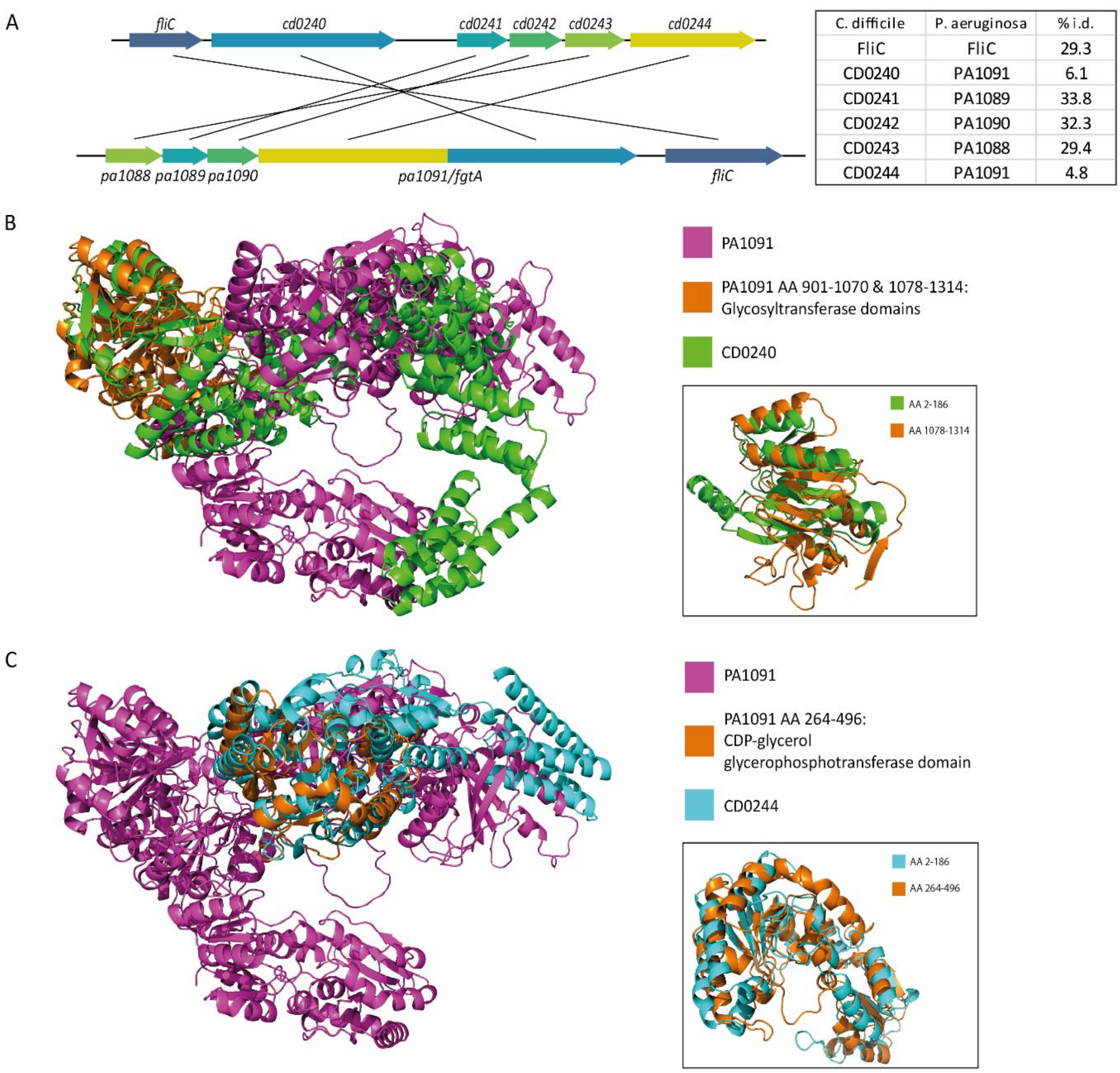

C. difficile	P. aeruginosa	% i.d.
FliC	FliC	29.3
CD0240	PA1091	6.1
CD0241	PA1089	33.8
CD0242	PA1090	32.3
CD0243	PA1088	29.4
CD0244	PA1091	4.8

Figure S1. PA1091 from *P. aeruginosa* contains multiple domains that are similar to both CD0240 and CD0244 from *C. difficile* 630Δ*erm*. A) A schematic representation of the FliC post-translational modification gene clusters in both *C. difficile* and *P. aeruginosa*. The lines connect the genes which products have similar predicted functions. The percentage of identities of the homologous proteins are shown in the table on the right. **B)** CD0240 superimposed on PA1091. The predicted glycosyltransferase domain of CD0240 (AA 2-186) maps to the predicted glycosyltransferase domains of PA1091 (AA 901-1070 and 1078-1314, in orange). Inset: The predicted glycosyltransferase domain of CD0240 and one of the predicted domains of PA1091. **C)** CD0244 superimposed on PA1091. The predicted CDP-glycerol glycerophosphotransferase domain of CD0244 (AA 244-486) maps to the predicted CDP-glycerol glycerophosphotransferase domain of PA1091 (AA 264-496, in orange). Inset: The predicted CDP-glycerol glycerophosphotransferase domains of PA1091 and CD0244.

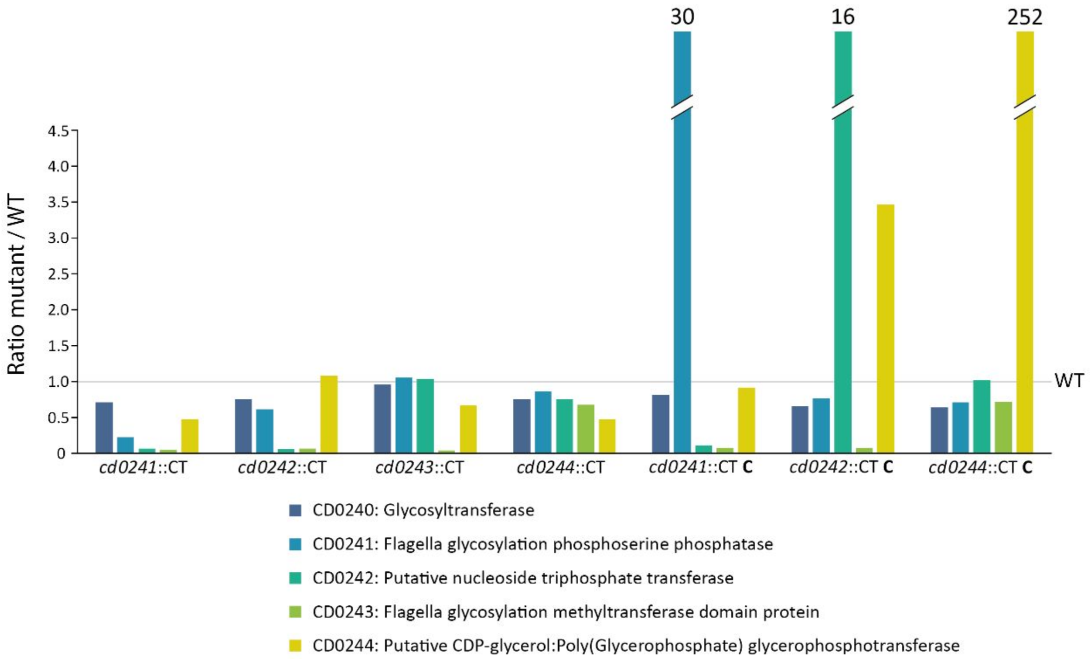

Figure S2. The relative levels of the Type A biosynthetic proteins in mutants with ClosTron insertions in the individual genes and their complemented strains. A quantitative proteomics experiment was performed using TMTpro 16plex labeling (each strain in duplicate). The protein levels of the Type A biosynthetic proteins in each of the individual strains relative to the WT are shown. For CD0241, CD0242, and CD0244 in their respective complemented strains, the ratios are depicted above the bars. Ratios are calculated based on the average absolute abundance of a protein from two replicates per strain.

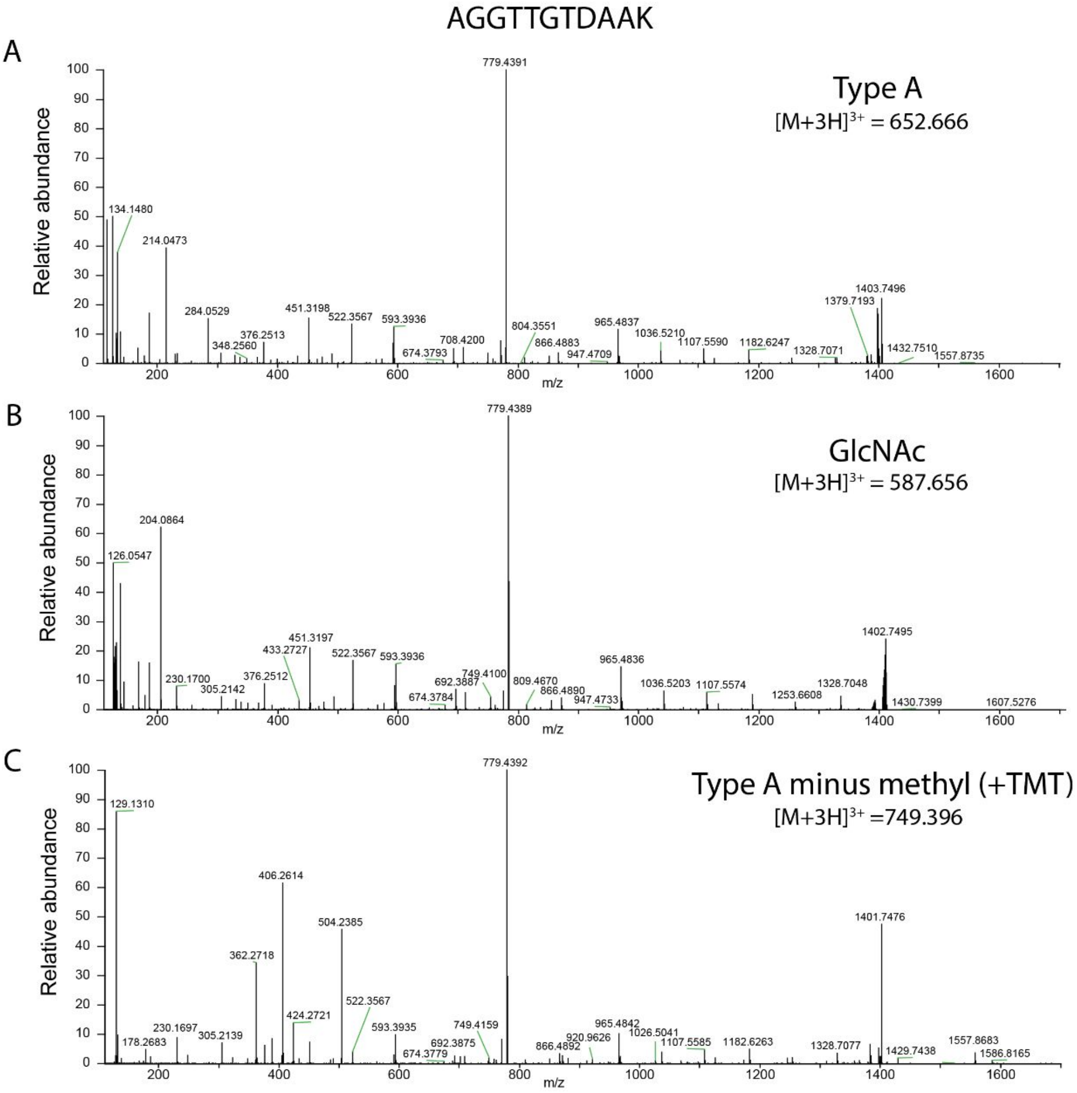

Figure S3. Summed MS/MS spectra of the AGGTTGTDAAK peptide displaying the Type A variants. Targeted HCD MS/MS analysis of the TMTpro 16plex labeled strains was performed. MS/MS spectra were summed over the full peak corresponding to the AGGTTGTDAAK peptides displaying the complete Type A (**A**), only the GlcNAc (**B**) or Type A minus the methyl group but having an extra TMT label (**C**). The theoretical precursor masses are shown on the right.

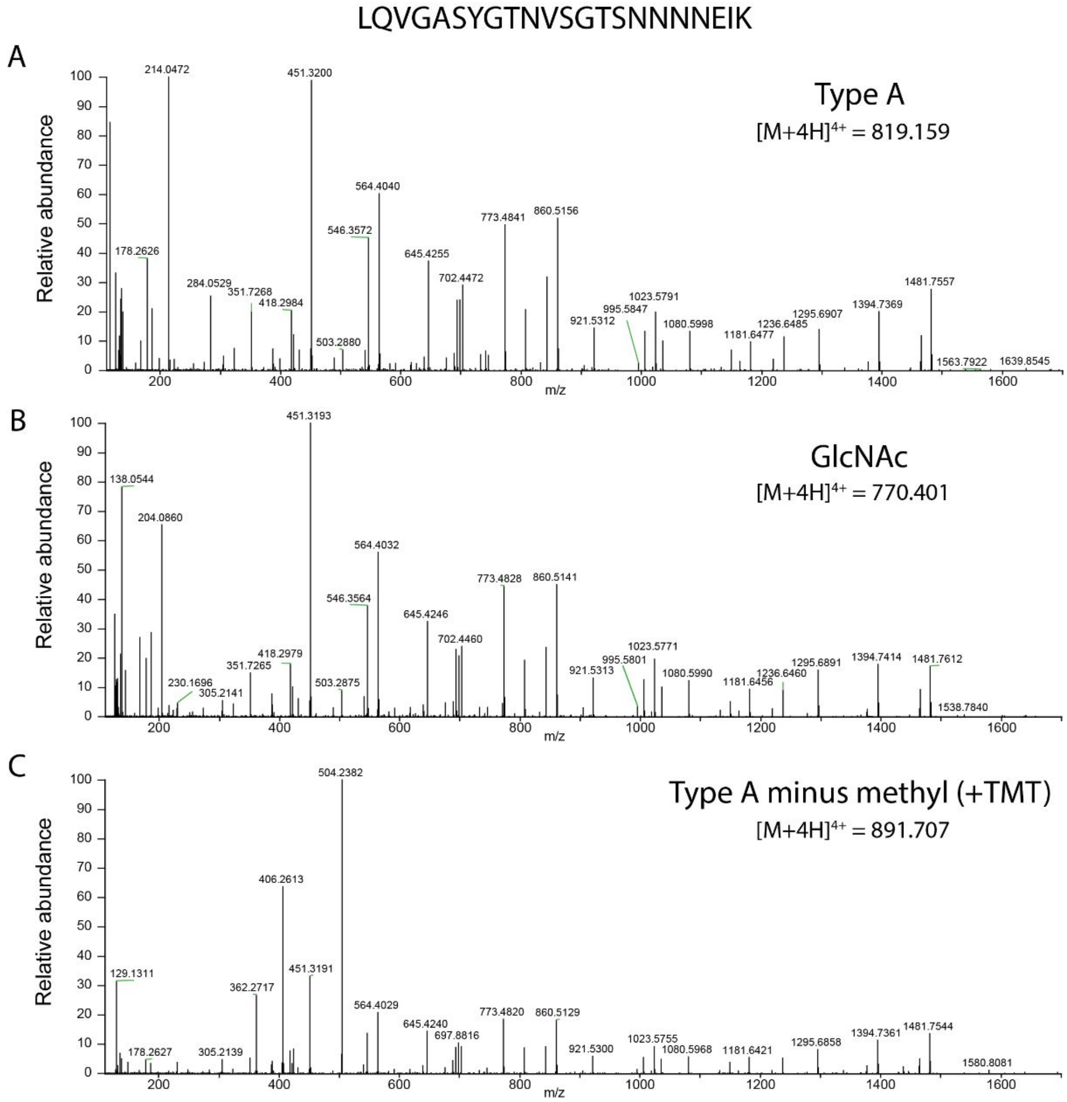

Figure S4. Summed MS/MS spectra of the LQVGASYGTNVSGTSNNNNEIK peptide displaying the Type A variants. Targeted HCD MS/MS analysis of the TMTpro 16plex labeled strains was performed. MS/MS spectra were summed over the full peak corresponding to the LQVGASYGTNVSGTSNNNNEIK peptides displaying the complete Type A (**A**), only the GlcNAc (**B**) or Type A minus the methyl group but having an extra TMT label (**C**). The theoretical precursor masses are shown on the right.

TMVSSLDAALK

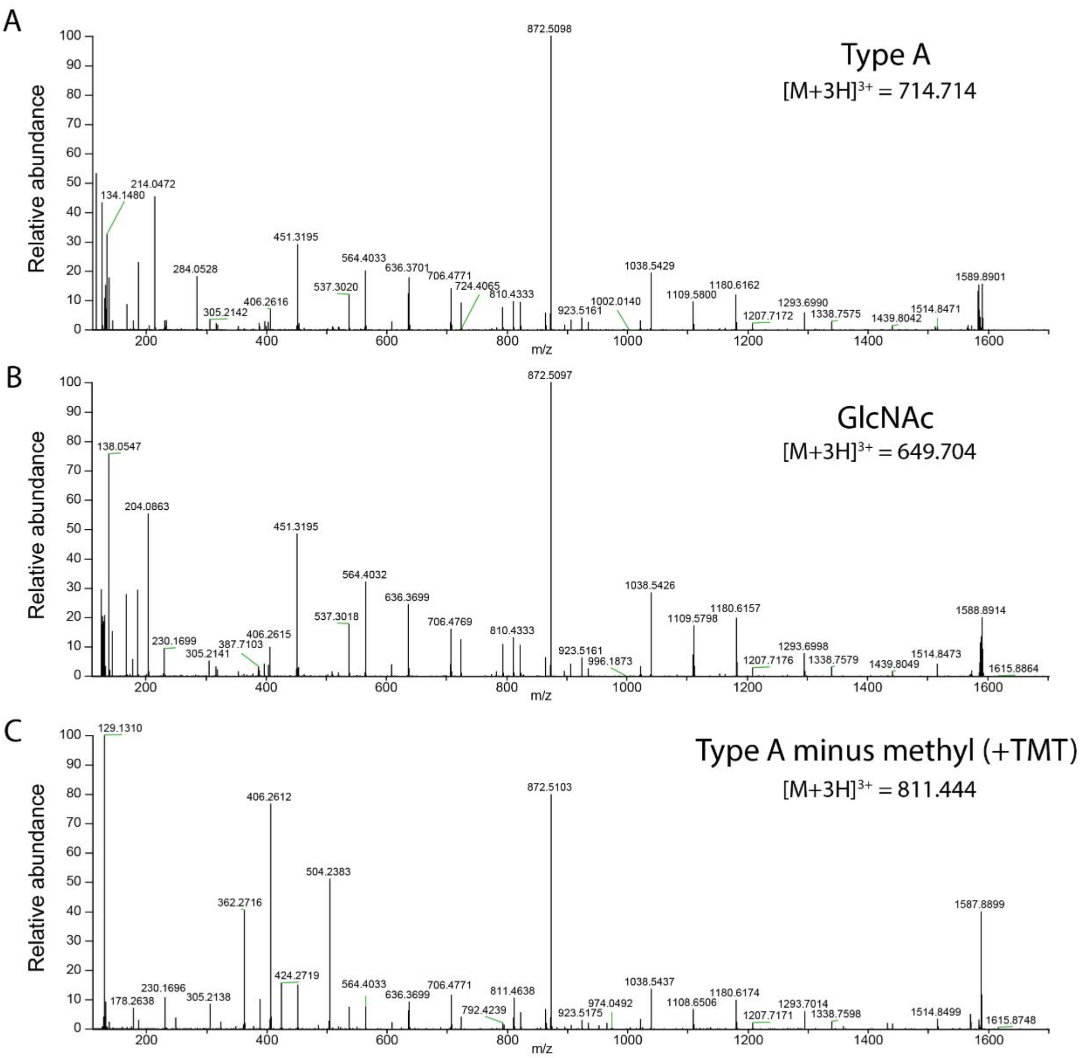

Figure S5. Summed MS/MS spectra of the TMVSSLDAALK peptide displaying the Type A variants. Targeted HCD MS/MS analysis of the TMTpro 16plex labeled strains was performed. MS/MS spectra were summed over the full peak corresponding to the TMVSSLDAALK peptides displaying the complete Type A (**A**), only the GlcNAc (**B**) or Type A minus the methyl group but having an extra TMT label (**C**). The theoretical precursor masses are shown on the right.

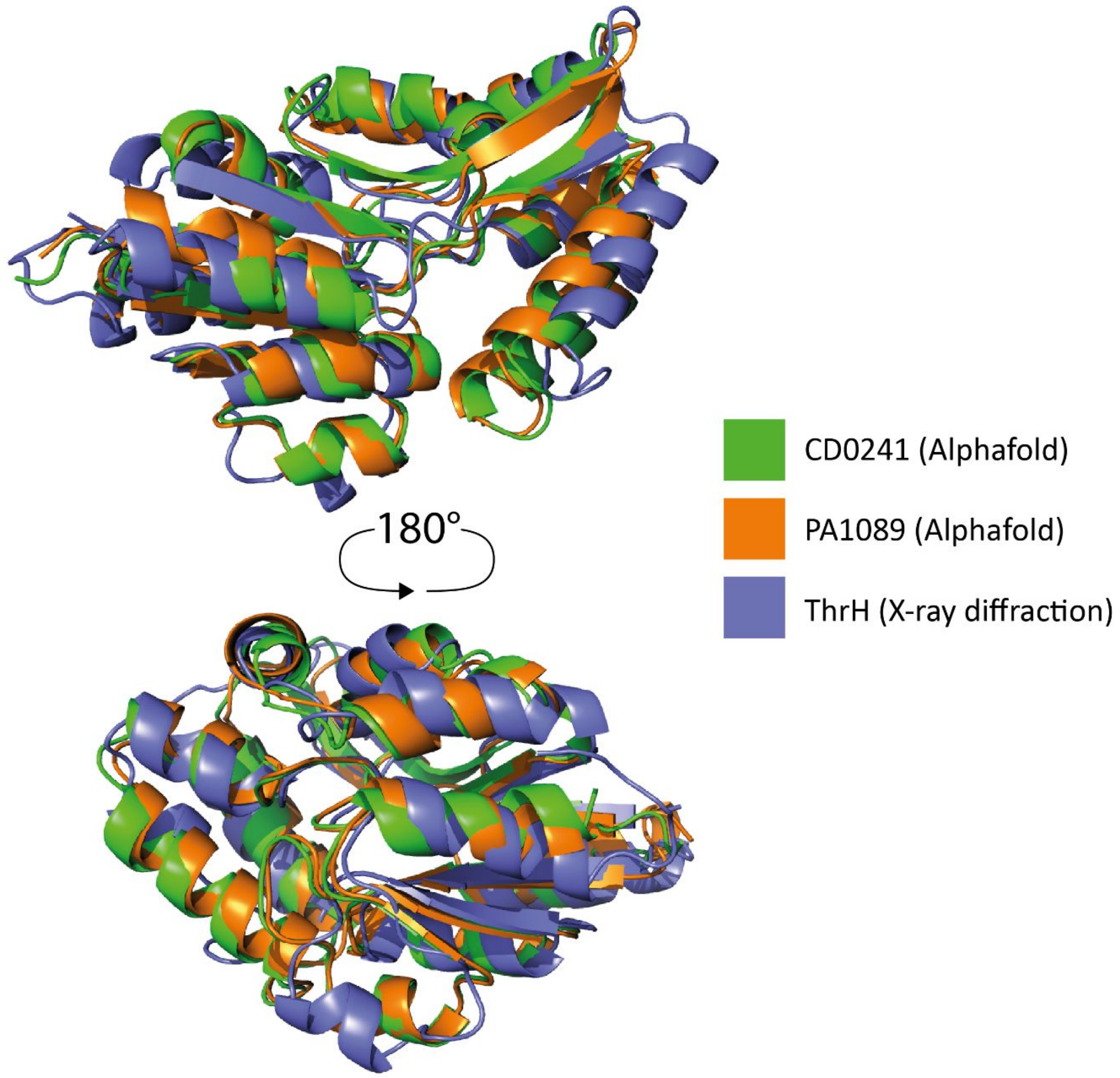

Figure S6. The predicted structures of CD0241 (*C. difficile*) and PA1089 (*P. aeruginosa*) are similar to the experimentally determined structure of ThrH from *P. aeruginosa*. The predicted structures for CD0241 and PA1089 were retrieved from the Alphafold database and superimposed on ThrH (PDB: 1RKU).

Table S2. Overview of the TMTpro labels per strain.

TMTpro label	TMT 16plex experiment	TMT 15plex experiment
126	WT	-
127N	*cd0241*::CT	WT
127C	*cd0241*::CT	WT
128N	*cd0242*::CT	WT
128C	*cd0242*::CT	*cd0241*::CT
129N	*cd0243*::CT	*cd0241*::CT
129C	*cd0243*::CT	*cd0241*::CT
130N	*cd0244*::CT	*cd0242*::CT
130C	*cd0244*::CT	*cd0242*::CT
131N	*cd0241*::CT comp.	*cd0242*::CT
131C	*cd0241*::CT comp.	*cd0243*::CT
132N	*cd0242*::CT comp.	*cd0243*::CT
132C	*cd0242*::CT comp.	*cd0243*::CT
133N	*cd0244*::CT comp.	*cd0244*::CT
133C	*cd0244*::CT comp.	*cd0244*::CT
134	WT	*cd0244*::CT
